# Toward reliable machine learning with *Congruity*: a quality measure based on formal concept analysis

**DOI:** 10.1007/s00521-022-07853-7

**Published:** 2022-10-06

**Authors:** Carmen De Maio, Giuseppe Fenza, Mariacristina Gallo, Vincenzo Loia, Claudio Stanzione

**Affiliations:** 1 Department of Computer Engineering, Electrical Engineering and Applied Mathematics, University of Salerno, 84084 Fisciano, SA Italy; 2 Department of Management and Innovation Systems, University of Salerno, 84084 Fisciano, SA Italy; 3 Defence Analysis and Research Institute, Center for Higher Defence Studies, 00165 Rome, RM Italy

**Keywords:** Machine learning, Black-box models, Explainable artificial intelligence, Interpretable machine learning

## Abstract

The spreading of machine learning (ML) and deep learning (DL) methods in different and critical application domains, like medicine and healthcare, introduces many opportunities but raises risks and opens ethical issues, mainly attaining to the lack of transparency. This contribution deals with the lack of transparency of ML and DL models focusing on the lack of trust in predictions and decisions generated. In this sense, this paper establishes a measure, namely *Congruity*, to provide information about the reliability of ML/DL model results. *Congruity* is defined by the lattice extracted through the formal concept analysis built on the training data. It measures how much the incoming data items are close to the ones used at the training stage of the ML and DL models. The general idea is that the reliability of trained model results is highly correlated with the similarity of input data and the training set. The objective of the paper is to demonstrate the correlation between the *Congruity* and the well-known *Accuracy* of the whole ML/DL model. Experimental results reveal that the value of correlation between *Congruity* and *Accuracy* of ML model is greater than 80% by varying ML models.

## Introduction

The cornerstone of how artificial intelligence (AI) works is ML—the ability of machines to learn from experience and evolve as they learn continuously. The explosion of ML research and applications has made AI what it is today in terms of interest, investment, and applications. ML is an AI application that allows systems to learn and improve from experience without automatically being programmed. The ML algorithm is the recipe for teaching the machine to learn, and the ML model is the result of the learning, which can then be generalized to new data. Whatever algorithm is used to create an ML model, there is one fundamental truth: it is only as good as its data. Bad data leads to bad models. Bad models are easy to spot because they perform poorly in many cases.

What happens if you are a user of a model that is not performing well? What happens if the model you are using performs poorly? Was it trained on the wrong data? Did the data scientists choose a selective or biased dataset that does not match their reality? Did they select incorrect hyper-parameters that may work well for the programmers but not for you?

Finding answers to these questions is almost impossible due to a lack of transparency in the ML or DL models. Since the market shifts from model builders to model users, more visibility and transparency make one able to trust the models that others built [1, 2]. Concerns related to model non-explicability can be multiple; firstly, the algorithm can be unexplainable in terms of outputs; secondly, there can be a lack of visibility of the training set. Such last aspect also determines opacity about data selection methods and bias in the training set that is also worsened by data constant changes due to drifts.

Designing a fully transparent model is a long and not easy task. EXplainable artificial intelligence (XAI) [3, 4] is an emerging trend related to the methods and techniques of applying AI technology.

The XAI is a set of methods and processes that allow users to understand and consider reliable results and output created by ML algorithms [5, 6]. It is used to describe an AI model, its expected impact, and potential errors. In addition, it helps to characterize the model precision, correctness, transparency, and results in the decision-making process with AI technology.

The US National Institute of Standards and Technology (NIST) [7] has developed four explainable AI principles that capture a variety of disciplines that contribute to explainable AI, including computer science, engineering, and psychology.

This work mainly impacts the following principle: *“The system should be able to explain its output and provide supporting evidence (at least)”*. To achieve greater trust in ML, it provides the following explanation types:

- Explanations that benefit the end-user;
- Explanations that are designed to gain trust in the system;
- Explanations that are expected to meet regulatory requirements;
- Explanations that can help with algorithm development and maintenance;
- Explanations that benefit the model owner, such as movie recommendation engines.

This contribution focuses on the lack of transparency enabling users to trust in predictions and decisions generated by the ML and DL models. The paper defines a *Congruity* measure to support the ML model explainability by enhancing the training dataset transparency. It is mainly devoted to providing reliability value every time we invoke an ML model after its creation. Like the Accuracy gives an idea of the performance of the model, the proposed Congruity is a value that says to the users how much the result provided by the ML model is reliable. Such an index is mainly helpful for whoever (different from the researcher who created it) adopts the model without knowing the training details. Indeed, let us suppose we are asking to classify an email as spam or not. According to our idea, the system will provide the resulting label (spam or not spam) and the corresponding reliability measure, i.e., Congruity. The Congruity measures how much the model is aware of items similar to the input one considering the dataset used during the training stage. The proposal gets inspiration from the out-of-distribution detection paradigm [8]. It aims to evaluate the level of representativeness of new instances concerning the training set, to assess learning model predictivity. Despite the high overall Accuracy, the Congruity stresses that if the model is low experienced with the input, the user cannot trust the resulting classification. Moreover, the researcher who is training an ML model may use the average of the Congruity measured on the test set to know whether the training set is well-balanced and how well it represents the problem data.

More in detail, *Congruity* is a function that can be applied to each data item input of the ML model. It is evaluated by browsing a lattice built on the training dataset using Formal Concept Analysis (FCA) [9]. The result of the *Congruity* function reveals how much the data item input of the ML model is well represented in the training dataset. The objective of this work is to verify that *Congruity* is correlated to the *Accuracy* of an ML model. Once the correlation is confirmed, *Congruity* could be used twofold. Firstly, it could be used at the training stage of the ML model to select the most representative training dataset by averaging the *Congruity* value of the test set. Secondly, *Congruity* could be used later to know the trustability of the ML model results when the model is running on new real inputs. Experimentation is mainly performed on three existing datasets by varying the ML techniques. It reveals a high correlation between *Congruity* and *Accuracy*.

The main contributions of this paper are:

- Definition of the *Congruity* as a measure for evaluating how much a data item is represented in a training dataset by organizing data in a lattice structure extracted using FCA theory.
- Evaluation of the correlation between the defined *Congruity* and the *Accuracy* of ML and DL models.
- Results of the correlation between *Congruity* and *Accuracy* evaluated on three public real-world datasets using the following ML algorithms: Kernel Support Vector Machine, Random Forest, Artificial Neural Network, Deep Neural Network, and Multilayer Perceptron.

The remainder of the manuscript is organized as follows. Section 2 presents the related works. Then, Sect. 3 introduces the math notation and the *Congruity* measure definition. Section 4 describes how this measure is evaluated on top of the lattice extracted using FCA. Section 5 provides the results in terms of correlation between *Congruity* and *Accuracy* on different models and datasets. Finally, conclusions and future works, in Sect. 6, argue possible applications of the *Congruity* measure for detecting drift and bias of ML models, a hot topic also from an ethical perspective.

## Related work

The research community is usually focused on designing complex models able to achieve a high level of prediction performance. Nevertheless, simple models (e.g., linear models) are often preferred for their ease of output interpretation, even if they may be less accurate than complex ones. However, simpler models are not always suitable, especially for solving complex problems. Complex problems, such as the exponential growth of big data, require complex models, thus leading to the trade-off between accuracy and interpretability of model output. In the literature, a wide variety of solutions have been proposed to try to reduce this issue.

Authors in [10] proposed *LIME*, an epic clarification strategy that learns an interpretable model locally around expectations. [11] presents *DeepLIFT* (Learning Important FeaTures), a technique for processing significance scores in a neural network. The paper in [12] presents a sensitivity analysis-based strategy for clarifying expectation models applied to an order or regression model. A proper establishment to improve the straightforwardness of decision-making frameworks is introduced in [13]. *SHAP* (Shapley Additive exPlanations) [14] allots each feature an importance value for a particular prediction. Caruana et al. [15] present two case studies where high-performance generalized additive models with pairwise interactions (GA^2^Ms) are applied to genuine medical services issues yielding coherent models with best-in-class precision. A decision list, modelled through a series of if/then statements (e.g., if hypertension, at that point stroke) that discretizes a high-dimensional, multivariate component space into a progression of straightforward, promptly interpretable choice proclamations, is proposed in [16]. Regarding training dataset transparency, the work in [17] evaluated the correlation between the proposed data consistency measure and Learning To Rank performances. Results demonstrated that the consistency of a training dataset (weighted by leveraging consensus of group decision making technique) heavily correlates to the accuracy of a DNN trained by the same dataset. Similarly, the work in [18] estimates prediction changes due to modifications in the adopted training set. Authors of [19] implement a density and local fit principle. The density principle measures the similarity between the new instance and the training set. The local fit principle measures the learning model performance on training subsets more similar to the instance under evaluation.

Another methodology for producing clarification is to fabricate an additional model over the outcome of a unique model. In this sense, Letham et al. [20] find a solution to the inquiry of the most probable mark of a given inconspicuous information point. In [21], a novel algorithm, TREPAN, is presented for removing fathomable, emblematic portrayals from trained neural networks. [22] proposes an overall answer for the issue of understanding characterization choices by pixel-wise decay of non-straight classifiers. In [23], a system that uses the formal concept analysis to explain artificial intelligence models is proposed. In addition, the authors use grouping examination to consider anomalies in the information, which is also used to clarify the result of the AI model. To the best of our knowledge, the work in [23] is the only one using the formal concept analysis to explain AI, which is the proper use of the FCA technique. However, in [23], FCA is used as a white-box classification model for implementing ML. In this work FCA is used to build the lattice and then calculate the value of the new Congruity index to say in advance how the ML model will behave.

In addition to the explainable machine learning paradigm, some researchers propose a learning model reliability assessment by approaching the out-of-distribution detection (OOD) problem. In particular, authors in [24] demonstrate that out-of-distribution examples are more likely to be erroneously classified; they identified them through the softmax distribution. The OOD problem has been often approached by training specific Neural Networks [25, 26]; however, such solutions could further hide measurement mechanisms without contributing to an Explainable solution as our approach aims to do.

## Congruity measure based on formal concept analysis

As outlined in the introduction, this work aims to find a correlation between machine learning and deep learning models and the *Congruity* concerning the dataset used during the training. The idea is to compare the *Accuracy* of the ML/DL model by varying the *Congruity* of items in the test set.

This section defines the *Congruity*. It measures how well a data item is represented in a data set (i.e., the training set). In the following subsections, the notation and the foundations of the theory are given before introducing the *Congruity* that is calculated by traversing the lattice-based structure extracted using formal concept analysis (FCA) theory.

The motivations for using the FCA and the resulting lattice for evaluating the *Congruity* are essentially the following ones: (i) coherently with the aim underlying this research work about the transparency of ML, FCA, and lattice are white-box data mining techniques; (ii) the lattice gives the opportunity to summarize data with different levels of granularity useful to empirically change the configuration of the *Congruity* evaluation according to the specific needs.

### Notation

The following is the essential notation used in the next subsections for defining the *Congruity*:

- *M* - set of context attributes;
- *G* - set of context objects;
- *L* - lattice resulting from FCA;
- Attr(*X*) - attributes of an object or a concept *X*.
- Objs(*C*) - objects of a concept *C*.
- $C_i = (A_i,B_i)$ - Concept $i-th$ where $A_i \equiv Attrs(C_i)$ and $B_i\equiv Objs(C_i)$ are the set of attributes and the set of objects of concept $C_i$, respectively.
- *c*(*L*, *New*)—Congruity function that, given a lattice *L* and a new instance (or object) *New*, associates a real value in $[0-1]$.
- $G'= \{G'^1,G'^2, ..., G'^k\}$ whose intersection cardinality $|Attr(New) \cap Attr(G'_i) |> 0$ for $i=1,2, ..., k$;
- *S*(*C*) - support of a concept *C* calculated as the ratio:
  $$\begin{aligned} S= \frac{\vert {\rm Objs}(C) \vert }{\vert G' \vert } \end{aligned}$$
- *P*(*C*, *New*) - given an input instance *New* and a concept *C*, the Precision *P* is evaluated as:
  $$\begin{aligned} P= \frac{\vert {\rm Attr}(New)\bigcap {\rm Attr}(C) \vert }{ \vert {\rm Attr}(C) \vert } \end{aligned}$$
- *R*(*C*, *New*) - given an input instance *New* and a concept *C*, the Recall *R* is evaluated as:
  $$\begin{aligned} R= \frac{\vert {\rm Attr}({\rm New}) \cap {\rm Attr}(C) \vert }{\vert {\rm Attr}({\rm New}) \vert } \end{aligned}$$
- $F-{\rm Measure}(C, {\rm New})$ - the combination of Precision and Recall evaluated between instance *New* and concept *C* is given by the following equation:
  $$\begin{aligned} F-{\rm Measure}= 2 * \frac{P * R}{P + R} \end{aligned}$$

### Formal concept analysis

The formal model behind the proposed methodology is the formal concept analysis (FCA) [9]. In the literature, the formal concept analysis (FCA) is known as a method for knowledge representation, information management and data analysis [27]. It is able to understand relationships between a set of objects and a set of attributes represented in the formal context (through a tabular way). So, it detects concepts containing objects sharing the same attributes. In this way, the resulting lattice represents the underlying structure of the analyzed context.

From its introduction, FCA was applied for numerous purposes, for example, data mining, data analysis, information retrieval, taxonomies and ontologies building, clustering, recommendation, network analysis [28–30], etc. More recently, it was also adopted for machine learning explainability goals [23].

Following, some definitions about FCA are given.

#### Definition 1

(*A Formal Context*) is a triple $K=(G,M,I)$, where *G* is a set of objects, *M* is a set of attributes, and $I= \left( G \times M \right)$ is a binary relation. $(g,m) \in I$ is read “object *g* has attribute *m*”.

The context is often represented as a “cross table” (see Table 1): the rows represent the formal objects and the columns are formal attributes; the relations between them are represented by the crosses.

Taking into account the formal context, FCA algorithm is able to identify Formal Concepts and subsumption relations among them. More formally, the definition of formal concept and order relation among them are given as follows:

#### Definition 2

(*Formal Concept.*) Given a formal context $K=(G, M, I)$, for ${A} \subseteq G$, apply a derivation operator, $A^{'}=\{m\in M\ \vert \ \forall g \in A:(g,m)\in I\}$ and for ${B}\subseteq M$, $B^{'}=\{g \in G \ \vert \ \forall m \in B:(g,m) \in I\}$. A formal concept *C* is identified with a pair $C=(A,B)$, where ${A} \subseteq G$, ${B}\subseteq M$, such that $A'=B$ and $B'=A$.

#### Definition 3

Given two concepts $C_1=(A_1,B_1)$ and $C_2=(A_2,B_2)$, then $C_1$ is a subconcept of $C_2$ (equivalently, $C_2$ is a superconcept of $C_1$), $(A_1, b_1) \le (A_2, B_2) \Leftrightarrow A_1\subseteq A_2 (\Leftrightarrow B_2 \subseteq B_1).$ The set of all concepts of a particular context, ordered in this way, forms a complete lattice.

**Fig. 1:**
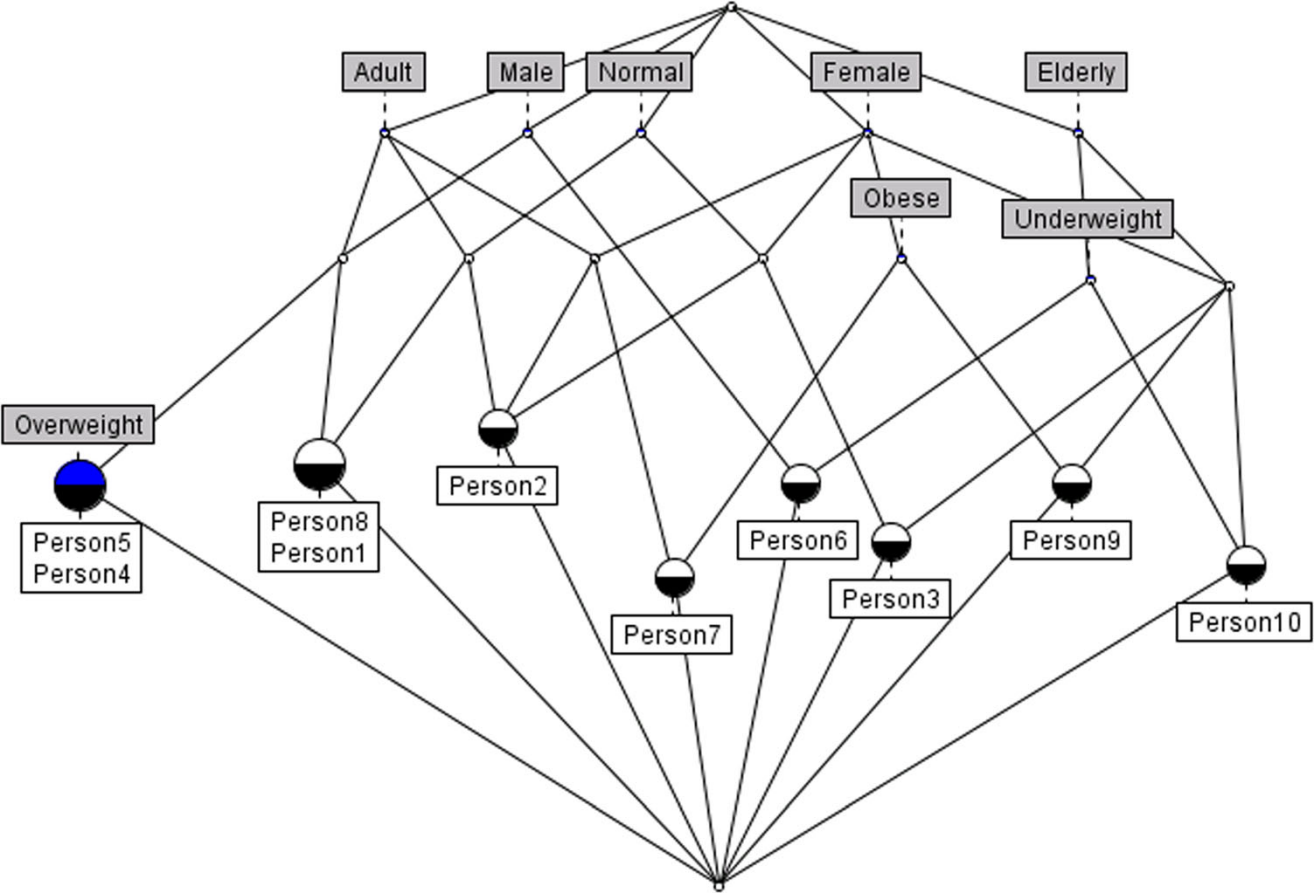
Lattice example—The figure shows the lattice generated from the formal context in Table 2. Each concept owns objects (i.e., people) that share the same attributes (e.g., *Person1* and *Person8* that share *Adult*, *Male*, and *Normal*)

Note that each node in Fig. 1 (i.e., a Formal Concept) comprises the objects and the associated set of attributes. In the figure, each node has a different color according to its characteristics: a half-blue colored node represents a concept with *own* attributes; a half-black colored node instead outlines the presence of *own* objects in the concept; finally, a half-white colored node represents a concept with no *own* objects (if the white-colored portion is the half below of the circle) or attributes (if the white half is up on the circle).

Given the Formal Concepts, it is easy to see that the subconcept relation $\le$ induces a *Lattice* of Formal Concepts. As a matter of fact the lowest concept contains all attributes and the uppermost concept contains all object of the Formal Context.

#### FCA example

Let us explain FCA through a practical example.

Assume to have information about age, gender, and Body Mass Index (BMI) of 10 people, as shown in Table 1.

**Table 1:** Example data—The table shows the attributes representing each person used to build the example

Person	Age	Gender	BMI
Person1	25	Male	23
Person2	59	Female	20.5
Person3	68	Female	24
Person4	18	Male	25
Person5	44	Male	27
Person6	81	Male	17.5
Person7	33	Female	31
Person8	49	Male	19.5
Person9	77	Female	30
Person10	90	Female	18

The construction of the formal context needs information bucketing. In particular:

- For categorical attributes, such as *gender*, we define an attribute for each possible value (i.e., *Male* and *Female*).
- For numerical attributes (i.e., *age* and *BMI*), we establish some thresholds and define an attribute for each range. In particular, people are considered *Adult* if their age is lower than 60; otherwise, *Elderly*. Regarding BMI, we assume:
  - *Underweight* people have a BMI lower than 18.5;
  - *Normal* people have a BMI between 18.5 and 24.9;
  - *Overweight* people have a BMI between 25 and 29.9;
  - *Obese* people have a BMI greater than or equal to 30.

The resulting Formal Context is one in Table 2. As notable, for each row (i.e., person) there is a “X” at each intersection with the owned attribute.

**Table 2:** Formal context example—After an appropriate bucketing process, the attributes shown in Table 1 create the necessary context for constructing the lattice

	Adult	Elderly	Male	Female	Under- weight	Normal	Over- weight	Obese
Person1	X		X			X		
Person2	X			X		X		
Person3		X		X		X		
Person4	X		X				X	
Person5	X		X				X	
Person6		X	X		X			
Person7	X			X				X
Person8	X		X			X		
Person9		X		X				X
Person10		X		X	X			

By applying the FCA algorithm, the lattice in Figure 1 results. We can notice that people with the same attributes (e.g., *Person4* and *Person5*) rely on the same concept.

### Congruity

FCA conceptualizes input data and generates a hierarchical knowledge structure (i.e., lattice *L*). *Congruity* is defined by the lattice resulting from the FCA. It is a function *c*(*L*, *X*) that takes as input the lattice *L* and a new data item, i.e., the instance *X*. Intuitively, the *Congruity* should represent a measure of representativeness of *X* in *L*, which describes qualitatively and quantitatively the modeling of the new input instance with the concepts already available in the extracted lattice. In essence, two pieces of information are evaluated:

- how many items with the same characteristics were in the sample set during the lattice construction;
- the coverage degree of characteristics of the new instance with concepts in the lattice *L*.

If we consider a machine learning classifier, the level of Congruity depends on how much the sample used to train the classification model is representative of new instances to classify. That happens, on the one hand, if the features of the classifier include the characteristics of the instance to classify, and, on the other hand, if the classification of similar objects is being trained a number of times such that the classes are balanced in terms of the number of considered instances. Starting from this observation, this work aims to evaluate the correlation between the proposed *Congruity* measure and the measure of reliability of a classifier output.

*Analysis of possible cases.* Given the previous intuitive definition of Congruity, we could say that in the presence of a new input object, there are two aspects to consider:

- the *support* of the concept(s) in which the new input instance would fall (or which are closest to that in which it would fall);
- the *coverage* of the input instance in terms of attributes in the available concepts. According to [31], the coverage could be the degree of matching calculated as the F-measure of new instance characteristics for the lattice concepts.

Thus, the *Congruity* could be a linear convex combination of the *support* and *F-measure* values between the characteristics of the concepts with a set of attributes whose intersection with the attributes of the new instance is non-empty. To assess the appropriateness of computing and combine these two values to obtain the *Congruity*, we proceed with the possible case enumeration.

Given a new instance *X* where ${\rm Attrs}(X)=\{a_1,a_2,..., a_n\}$, the cases that can occur with respect to the existing lattice *L* are the following:

1. There are one or more concepts in the lattice, $\widetilde{C}= \{\widetilde{C}^1,\widetilde{C}^2, ..., \widetilde{C}^k$} whose intersection cardinality $|{\rm Attr}(X) \cap {\rm Attr}(\widetilde{C}_i) |> \frac{|{\rm Attr}(X) |}{2}$ for $i=1,2, ..., k$, and then we have the following further subcases:
  Among all concepts in $\widetilde{C}$, there exists $\widetilde{C}^*$ such that Attrs($\widetilde{C}^*$) coincides with Attrs(*X*); in that case, the F-Measure($\widetilde{C}^*, X$) shall be maximum (i.e., equal to 1), and the Congruity will only have to consider the support of the concept $\widetilde{C}^*$. Assuming Congruity is a linear convex combination of the support and F-Measure values with weights $\alpha _1$ and $\alpha _2$, then we will have:
    1 $$\begin{aligned} \begin{matrix} \alpha _1 S(\widetilde{C}^*) + \alpha _2 F-Measure (\widetilde{C}^*,X) =\\ \alpha _1S(\widetilde{C}^*) +\alpha _2\\ \end{matrix} \end{aligned}$$
  In other cases, Congruity *c*(*L*, *X*) can be calculated as:
    2 $$\begin{aligned} \begin{matrix} c(L,X) = \frac{ 1}{\vert \widetilde{C} \vert } \sum _{\widetilde{C}^i \in \widetilde{C}} \alpha _1S(\widetilde{C}^i) +\\ \alpha _2 \ F-{\rm Measure} (\widetilde{C}^i,X)\\ \end{matrix} \end{aligned}$$
2. There is no concept $C^*$ such that the intersection cardinality $|Attr(X) \cap Attr(\widetilde{C}_i) |> \frac{|Attr(X) |}{2}$. In this case, the Congruity is $c(L,X)=0$.

### Congruity example

Let us start from the example in Sect. 3.2.1 and assume to try to classify two new people characterized as in Table 3. First, we need to evaluate its Congruity concerning the existing Lattice (i.e., Fig. 1) through a bottom-up visit for each new instance.

**Fig. 2:**
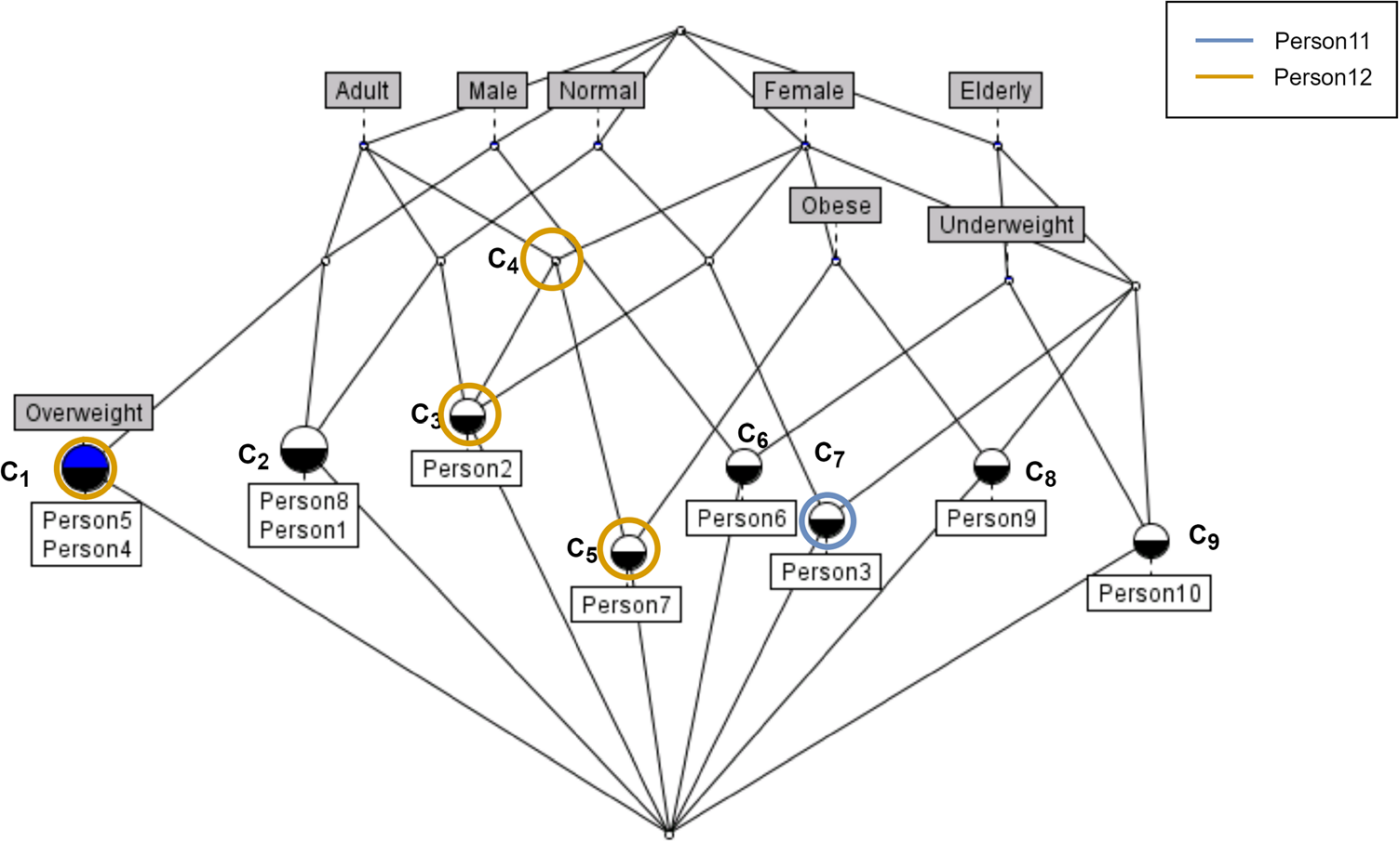
Congruity computation example—In the lattice, all generated concepts are represented; note that only those where objects are present or which match the example have been numbered to make the picture clearer. Moreover, in blue is highlighted the concept that completely matches *Person11*; in yellow, there are concepts matching *Person12*’s attributes. In particular, we only consider those concepts where the intersection with the attributes of the incoming instance is greater than half of the attributes for the new instance itself

Regarding *Person11*, we found a concept ($C_7$ in Fig. 2) that contains all new instance attributes. The Congruity index evaluation is as follows:

- The Support (the number of objects belonging to the retrieved concept divided to the total number of objects sharing at least one *Person11* attributes) measures $1/8 = 0.125$.
- The F-Measure is maximum (i.e., 1) because the concept attribute set corresponds with the instance attribute set.
- Let the weights $\alpha _1 = 0.1$ and $\alpha _2 = 0.9$, by following Equation 1, the *Congruity* value for *Person11* is: (0.125*0.1)+(1*0.9) = **0.913**.

**Table 3:** Formal context example of new items—The table presents attributes of new objects

	Adult	Elderly	Male	Female	Under- weight	*Normal	Over- weight	Obese
Person11		X		X		X		
Person12	X			X			X	

The second object, *Person12*, has the attribute set {adult, female, overweight}. This combination of attributes is new, and no concept like this already exists. So, the first step is to identify the concepts with the most similar set of attributes (i.e., concepts owning at least half of instance attributes). In this case, matching concepts are $C_1$, $C_3$, $C_4$, and $C_5$. Congruity evaluation starts from Support and F-Measure of these matching concepts, as shown in Table 4.

**Table 4:** Example Congruity computation—The table shows for each matched concept the results of support, precision, recall and then F-measure for the new Person12 instance

Concept	Support	Precision	Recall	F-measure
$C_1$	0.22	0.67	0.67	0.67
$C_3$	0.11	0.67	0.67	0.67
$C_4$	0	1	0.67	0.8
$C_5$	0.11	0.67	0.67	0.67

From Table 4, it follows that:

$$\begin{aligned} Congruity = \frac{1}{4} [(0.1 * 0.22) + (0.9 * 0.67) + (0.1 * 0.11) + \\ (0.9 * 0.67) + (0.1 * 0) + (0.9 * 0.8) + \\ (0.1 * 0.11) + (0.9 * 0.67) ] = \\ \frac{1}{4} [0.022 + 0.603 + 0.011 + 0.603 + 0.72 + 0.011 + 0.603 ] = \\ \frac{1}{4} * 2.573 = {\textbf {0.643}} \end{aligned}$$

The example serves to clarify the Congruity value and its calculation. It is good to consider that the example uses a minimal context and test set. After creating the lattice, we calculated the Congruity for the two new incoming instances, which in the specific case represent two people. In the case of *Person11*, the Congruity calculation is straightforward. The new instance completely matches an existing object. Therefore, the F-measure is equal to 1, as specified in the formulas previously. On the other hand, the Support is equal to 0.11 since there are eight objects involved in terms of attributes intersection. The Congruity for *Person11* is, therefore, equal to 0.913. Such a high value is due to the existence of the same attribute set in the initial context.

For *Person12*, the calculation involves four concepts considering an intersection cardinality between attributes of the new instance and once of the concept greater than half of the first one. Regarding the Support evaluation, we must consider 9 objects. Summing up, in this case, the Congruity value is 0.643. A good value, but not excellent, confirming that the learning model never saw these attributes together but only partially and with low Support. Of course, as mentioned before, there is to keep in mind that the example is based on a very low number of instances and could mislead the reader. On the other hand, the lattice can present millions and give rise to a profound representation of what is the context used to create it.

## Correlation between Congruity and accuracy

Explainability in Artificial Intelligence could refer to two aspects:

1. *Explaining the AI model pedigree*: how the model was trained, which data was used, which types of bias are possible, and how to mitigate them.
2. *Explaining the overall model*: this is also called “model interpretability.”

This work focuses on the first point: explaining the AI model pedigree. In particular, to explain the model pedigree means to answer the following questions:

- How was the model trained?
- What data was used?
- How was the impact of any bias in the training data measured and mitigated?

These questions are the data science equivalent of explaining what school your surgeon went to - along with who their teachers were, what they studied, and what grades they got. Of course, getting this right is more about the process and leaving a paper trail than pure AI, but it is critical to establishing trust in a model.

**Fig. 3:**
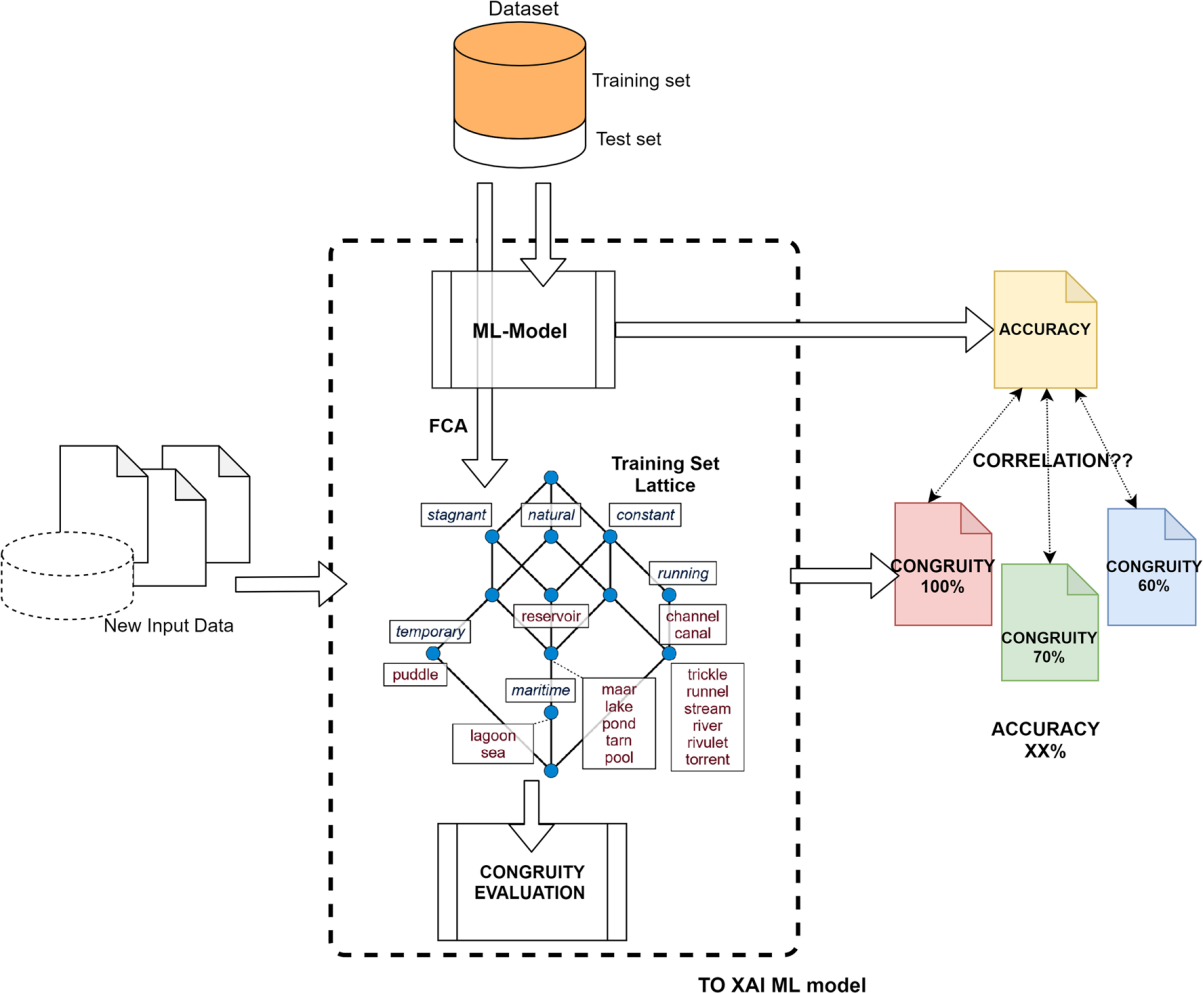
General workflow—The workflow reflects the general idea. First, the lattice is built using the entire training set. Then, the Congruity is calculated for each instance of the test set. The Congruity values are sorted and grouped by similar values. At this point, the model accuracy is calculated for each group. The aim is to demonstrate a correlation between the Congruity and accuracy values: Do increasing Congruity values correspond to increasing accuracy values?

Figure 3 shows the workflow of the proposed solution. The process starts with data pre-processing to obtain a dataset that allows the lattice extraction through the Formal Concept Analysis (FCA). Then, the following steps are carried out:

1. Lattice extraction of the training set by applying the FCA;
2. ML/DL model training on the same dataset;
3. Calculation of the test set Congruity concerning the lattice constructed on the training set;
4. Grouping Congruity values and calculating corresponding ML Accuracy;
5. Calculation of Congruity-Accuracy correlation.

The following subsections will detail each workflow step.

### Lattice extraction

This phase computes the FCA algorithm from the dataset used to train the learning model. The objective is to construct the formal lattice functional to calculate the *Congruity* indicator.

### ML/DL model training

In this phase, the dataset used for lattice construction is used to train the ML/DL model. The choice of implementing a classifier as a machine learning model is dictated by the fact that the lattice only serves to “measure” the dataset and not to make any prediction.

### Congruity computation

After creating the lattice through the training set, the Congruity value is calculated for each instance in the test set. The objective is to evaluate consistency between training and test sets.

### Grouping Congruity values and accuracy calculation

This step is fundamental for the final one, where the correlation between Congruity and accuracy of the classifier is calculated. The idea is to sort, in ascending order, Congruity values previously evaluated. Subsequently, group them to have a similar number of instances for each group. The grouping is done for values that are very close to each other.

### Calculation of Congruity-accuracy correlation

The last step is to calculate the correlation between Congruity and accuracy values. For this calculation, the values obtained in the previous step are used, where groups of instances with close Congruity values are associated with Accuracy values obtained using the classifier. Pearson’s correlation coefficient, Kendall’s Tau coefficient and Spearman’s rank correlation coefficient [32] are used to measure the correlation.

## Experimentation

This section describes experimentation conducted on two health datasets and a textual one. It discusses the achieved correlation between Congruity and ML/DL model classification accuracy.

### Tools

The main tools adopted during the experimentation are: a custom developed library implementing formal concept analysis (FCA) derived by extending “Colibri”^1^ Apache Solr^2^, used for indexing concepts hierarchy extracted by FCA; the sci-kit learn library^3^, used to construct and adopt the logistic regression model, keras^4^ library for ANN and DNN models building.

### Datasets

Since model interpretability plays a crucial role in decision support systems, especially in areas like health, the framework evaluation starts from two health datasets: Pima Indians diabetes database^5^(PIDD) and stroke prediction dataset^6^(SPD). In addition, experimentation on a textual dataset is conducted through the Coronavirus tweets NLP dataset^7^(CT).

Pima Indians diabetes database is originally from the National Institute of Diabetes and Digestive and Kidney Diseases. The objective of the dataset is to predict whether or not a patient has diabetes diagnostically.

Stroke prediction dataset is used to predict whether a patient is likely to get a stroke based on his/her characteristics like gender, age, other diseases, etc.

Coronavirus tweets NLP dataset collects tweets with a common topic, the coronavirus. the focus of the dataset is sentiment analysis with 5 labels ranging from “extremely positive” to “extremely negative”.

### Workflow

**Fig. 4:**
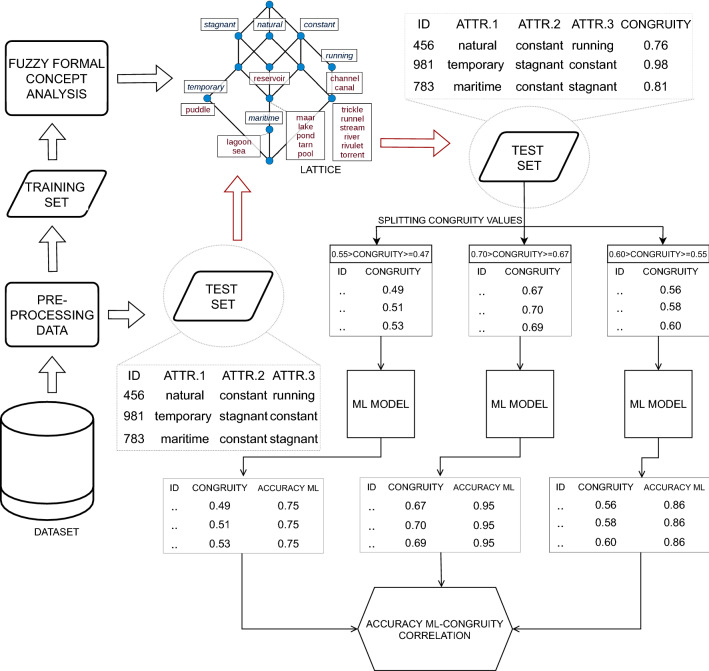
Experimentation workflow—The data is pre-processed and divided into training and test sets. In the case of the textual dataset, vectorization with Tf-idf was also adopted during the pre-processing phase. The formal concept analysis was applied to the training set, producing a lattice containing concepts representing data used to train the ML and DL models. The test set data is synthesized into queries to calculate the Congruity values for each incoming instance. The Congruity values are divided into equally distributed ranges. The corresponding instances are fed to the model to estimate the related accuracy. Finally, the correlation value between the two measures is calculated at the end of the process

In Fig. 4, the experimentation workflow is presented. It summarizes the application of the proposed methodology by reporting some application examples.

### Data preparation

Since the stroke prediction dataset had 201 samples with absent BMI (body mass index) value, rather than imputing it naively with the mean or median, we adopted a solution also suggested in the literature [33] that uses a decision tree model. The model establishes a fair decision tree model based on the age and gender of all other samples predictions for the missing values. Then, the dataset is divided into training and testing, with a percentage of 75–25% with 3832 and 1278 instances, respectively. The same percentage has been adopted for the PIDD dataset.

Regarding the textual dataset (i.e., CT) that consists of approximately 45,000 rows corresponding to likewise tweets, the pre-processing mainly applies a natural language processing workflow. It removes unnecessary parts (e.g., links, stopwords, users’ tags, etc.) and tokenizes the tweet textual content. Moreover, a vectorization was applied by exploiting the Tf-idf (term frequency-inverse document frequency) was applied. Finally, the dataset was divided into training and test sets with a percentage assigned to the latter of $30\%$, thus obtaining 31,352 instances in the training set and 13,338 in the test set.

### Experimental results

Four different models are used for experimenting with correlation among Congruity and accuracy values in the health datasets: a kernel support vector machine with a radial basis function kernel, a random forest with ten trees and entropy for the information gain, an artificial neural network (ANN) and a deep neural network (DNN), both using a sequential model fitted in 100 epochs. In DNN, hidden layers are 3. Two models were used for experimentation for the textual dataset, coronavirus tweets NLP, the random forest and the multilayer perceptron (MLP). Table 5 shows the accuracy values of each model on the overall test set for the three datasets.

**Table 5:** Overall models accuracy—The table shows the accuracy results of each adopted model

Dataset	Model	Accuracy
SPD	Deep neural network	$95.1\%$
	Kernel support vector machine	$95.2 \%$
	Random forest	$95\%$
	Artificial neural network	$94.7\%$
PIDD	Deep neural network	$93.9\%$
	Kernel support vector machine	$94.3 \%$
	Random forest	$93.6\%$
	Artificial neural network	$92\%$
CT	Multilayer perceptron	$84.3\%$
	Random forest	$81.9 \%$

Once the various *Congruity* values are calculated, the next step is adopting the selected ML/DL model with the corresponding instances. The objective is to demonstrate that a lower *Congruity* value corresponds to a lower *Accuracy* value and vice versa for the learning model.

**Fig. 5:**
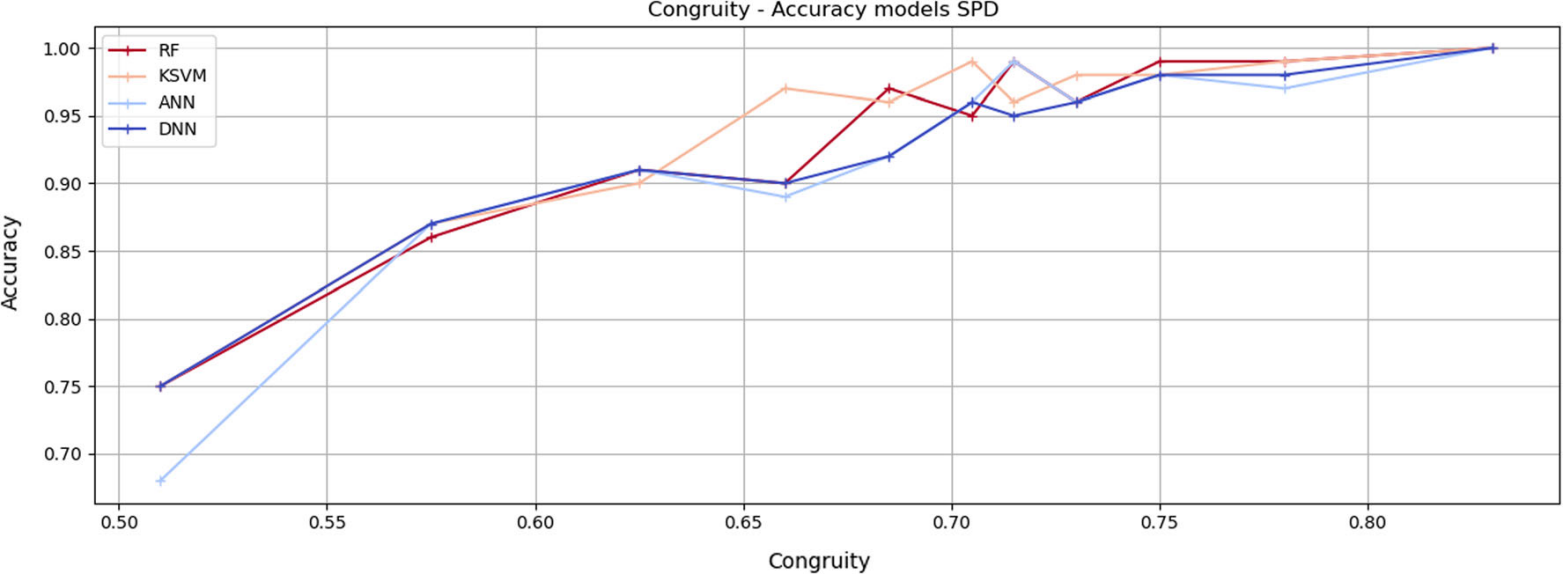
Congruity values range—Accuracy models on SPD dataset. The chart shows the accuracy values of the model tested with instances falling within the specific Congruity range. RF refers to random forest, KSVM to kernel support vector machine, ANN to artificial neural network and DNN to deep neural network

**Fig. 6:**
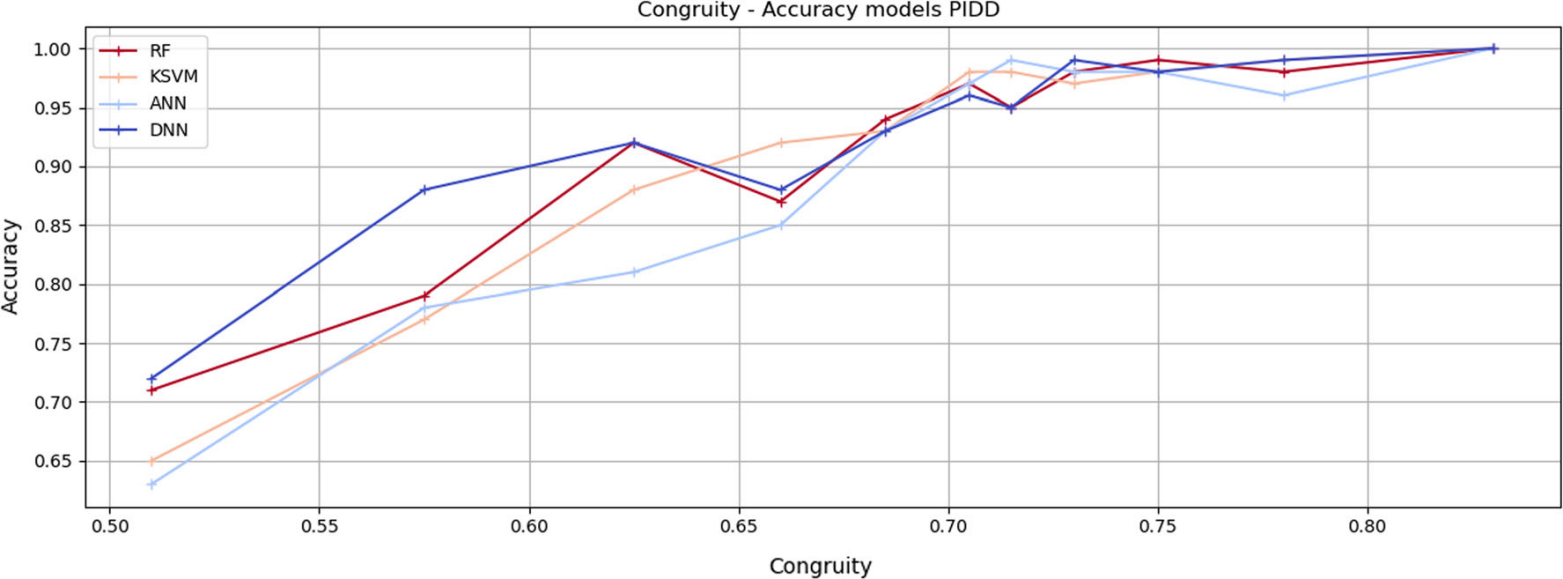
Congruity values range—Accuracy models on PIDD datasets. The chart shows the accuracy values of the model tested with instances falling within the specific Congruity range. RF refers to random forest, KSVM to kernel support vector machine, ANN to artificial neural network and DNN to deep neural network

**Fig. 7:**
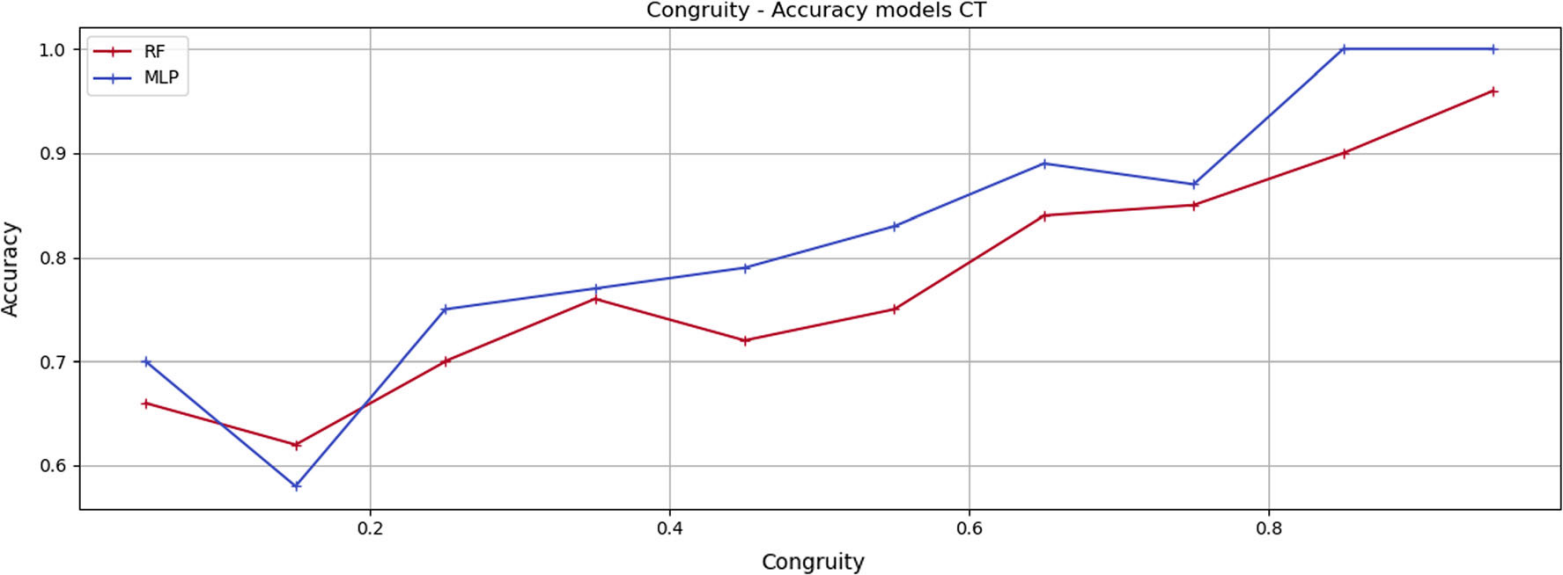
Congruity values range—Accuracy models on CT dataset. The chart shows the accuracy values of the model tested with instances falling within the specific Congruity range. MLP refers to multilayer perceptron and rf to random forest

Figures 5, 6 and 7 show the *Accuracy* values achieved by the adopted learning models for every group of instances in the specific *Congruity* range for each considered dataset. It is easy to notice that the increase in *Congruity* corresponds to an *Accuracy* increase.

The final step consists of calculating the correlation between the accuracy of the ML model and the Congruity values.

As shown in Table 6, in the SPD dataset, the best correlation for the random forest is 85.5%, 86.4% for KSVM, 86.1% for DNN, and 84.7% for ANN. Analogous results are achieved through the PIDD dataset. Better results are achieved through the Coronavirus Tweets dataset with a correlation of up to 92%.

**Table 6:** Correlation Congruity—Accuracy models. The table shows the correlation between Congruity and accuracy results of ML and DL models for respective datasets

Dataset	Model	Pearson	Kendall	Spearman
SPD	Deep neural network	$85.2\%$	$83.1\%$	$86.1\%$
	Kernel support vector machine	$85 \%$	$82\%$	$86.4\%$
	Random forest	$84.2\%$	$82.3\%$	$85.5\%$
	Artificial neural network	$83.7\%$	$82.5\%$	$84.7\%$
PIDD	Deep neural network	$84.9\%$	$82.1\%$	$85.1\%$
	Kernel support vector machine	$83 \%$	$81.3\%$	$83.2\%$
	Random forest	$84\%$	$82.3\%$	$84.5\%$
	Artificial neural network	$83.5\%$	$81.8\%$	$84.3\%$
CT	Multilayer perceptron	$89.4\%$	$87.8\%$	$92.1\%$
	Random forest	$88 \%$	$85.1\%$	$91.5\%$

### Comparison with state-of-the-art approaches

Although to the best of our knowledge, there not exist other similar indexes for measuring the reliability of a learning model, we tried to compare our proposal with an existing similarity measure. The objective is to understand whether a correlation between the train and test sets similarity and model Accuracy exists. In this sense, the Cosine similarity has been adopted. Cosine similarity is a metric for comparing two numerical sequences. Sequences are considered vectors in inner product space, and Cosine similarity is defined as the cosine of the angle between them, defined as the dot product of the vectors divided by the product of their lengths. During experiments, we evaluate the similarity between new instances and instances of the training set. Then, we evaluate the correlation between the mean Cosine similarity and accuracy. Table 7 shows the results. The reported correlations are lower than those evaluated through our proposed Congruity index. Moreover, a significant execution time is requested.

**Table 7:** Correlation Cosine similarity—Accuracy models. The table shows the Correlation between Cosine similarity and accuracy results of ML and DL models for respective datasets

Dataset	Model	Pearson	Kendall	Spearman
SPD	Deep neural network	$81.3\%$	$78.4\%$	$82\%$
	Kernel support vector machine	$81.8 \%$	$75\%$	$82.4\%$
	Random forest	$80.2\%$	$73.3\%$	$83.5\%$
	Artificial neural network	$79.5\%$	$70.5\%$	$82.4\%$
PIDD	Deep neural network	$79.3\%$	$72.4\%$	$79.5\%$
	Kernel support vector machine	$83.8 \%$	$78.1\%$	$84.6\%$
	Random forest	$82.2\%$	$76.3\%$	$81.3\%$
	Artificial neural network	$77.3\%$	$73.7\%$	$76.4\%$
CT	Multilayer perceptron	$80.3\%$	$61\%$	$78.2\%$
	Random forest	$78.4 \%$	$57.9\%$	$78.1\%$

### Discussion

From experimentation emerges a strong correlation between the proposed Congruity index and the performance of adopted ML and DL models. It follows that, by knowing the Congruity value, it is possible to deduce the reliability of the training set and, so, of the trained model giving more transparency to anyone who uses the model itself.

By comparing our approach with existing ones, we demonstrate that the Congruity has a higher correlation with the model Accuracy, which can guarantee more relevance during the reliability evaluation of the model. Moreover, although Congruity needs the lattice construction, it is done only the first time; subsequently, the index evaluation is converted into a query to a NoSQL database (i.e., Apache Solr) which quickly returns the best matching lattice concepts. On the contrary, a similarity-based approach (e.g., Cosine Similarity) must be evaluated for each test instance against each instance of the training set, requiring significant processing time.

Some limitations of the proposal regard the lack of experimentation on higher-dimensional datasets like images. In this sense, to reduce the FCA complexity, the literature suggests techniques like clustering or Linear Discriminant Analysis to group common characteristics and reduce the number of Formal Context attributes [34, 35].

## Conclusion and future works

The main objective of this research work is to introduce a methodology trying to give a measure of training set reliability to go toward more transparent and explainable ML models. In particular, this work proposes the *Congruity* indicator that, leveraging the Formal Concept Analysis, gives a qualitative and quantitative measure of the adopted dataset. The experimentation was carried out to compare different levels of Congruity against Accuracy achieved by different ML/DL models. A high correlation between these two values allows understanding of the model outcomes and, therefore, greater transparency. In this sense, experimental results are promising: when Congruity grows, the Accuracy grows in turn and vice versa. In particular, the correlation between Congruity and Accuracy is higher than one evaluated through the Cosine Similarity in all tests. So, we can conclude that, through the Congruity, we can say a priori how the model will behave concerning the training set used at the training stage.

Among possible future works, it could be interesting to predict when an existing learning model should be updated due, for example, to continue low levels of Congruity of new items. In this sense, the framework could also be adapted to recognize concept drifts in continuously evolving situations.

## Data Availability

Adopted datasets come from the following public domain resources: https://www.kaggle.com/uciml/pima-indians-diabetes-database. https://www.kaggle.com/fedesoriano/stroke-prediction-dataset. https://www.kaggle.com/datasets/datatattle/covid-19-nlp-text-classification.

## Footnotes

1 https://code.google.com/p/colibri-java/.

2 https://en.wikipedia.org/wiki/Apache_Solr.

3 https://scikit-learn.org/stable/.

4 https://keras.io.

5 https://www.kaggle.com/uciml/pima-indians-diabetes-database.

6 https://www.kaggle.com/fedesoriano/stroke-prediction-dataset.

7 https://www.kaggle.com/datasets/datatattle/covid-19-nlp-text-classification.
